# An FDG-first functional imaging approach in phaeochromocytoma and paraganglioma

**DOI:** 10.3389/fendo.2026.1833759

**Published:** 2026-07-21

**Authors:** Norah Alhazzaa, Mohammad M. R. Eddama, Fawziah Alorfi, Teng Teng Chung, Helen Simpson, Srirangalingam Umasuthan, Steve Hurel, Virginia Rozalen-Garcia, Tom Kurzawinski, Simon Wan, Jamshed Bomanji, Mark Gaze, Tarek Ezzat Abdel-Aziz

**Affiliations:** Department of Targeted Intervention, Division of Surgery and Interventional Science, University College London, London, United Kingdom

**Keywords:** paraganglioma, phaeochromocytoma, cancer, SDHB, functional scans, ^18^F-FDG PET CT/MRI, metastases

## Abstract

**Purpose:**

Functional imaging is central to the diagnosis and management of phaeochromocytomas and paragangliomas (PPGLs). This study aimed to compare the diagnostic performance of different functional imaging modalities for the detection of primary and metastatic PPGL.

**Methods:**

We conducted a retrospective, cross-sectional comparative study at a tertiary referral centre (2012–2023). Seventy three patients were with diagnosed with PPGL; 38 who underwent triple functional imaging. These 38 patients underwent ^18^F-FDG PET CT/MRI (FDG), and ^68^Ga-DOTATATE PET/CT (Dotatate), and ^123^I-mIBG SPECT/CT (mIBG). Per-patient detection rates for primary and metastatic disease were compared and correlated with SUV_max_, metanephrine profiles and germline mutations.

**Results:**

Among the 38 patients who underwent triple functional imaging, 18 (47%) were women, with a median age of 41 years (range 11-81). There were 23 phaeochromocytomas and 15 paragangliomas. Overall detection rates were positive in 36/38 (94.7%) patients on FDG, 35/38 (92.1%) on Dotatate, and 34/38 (89.5%) on mIBG. Among patients with metastatic disease, lesion detection was observed in 16/16 (100%) with FDG, 12/13 (92.3%) with Dotatate, and 11/13 (84.6%) with mIBG. FDG SUV_max_ correlated significantly with plasma 3-methoxytyramine levels (r = 0.56, p = 0.0034) but not with normetanephrine or metanephrine. FDG demonstrated 100% detection in patients with SDHB (6/6) and VHL (3/3) mutations.

**Conclusion:**

In this study, FDG demonstrated the highest per-patient detection and the strongest association with aggressive biochemical and genetic phenotypes. These findings support an FDG-first functional imaging strategy for diagnosis and staging in PPGL, with additional targeted imaging reserved for theranostic decision-making.

## Introduction

Abdominal paragangliomas and phaeochromocytomas (PPGLs) are rare catecholamine-secreting tumours arising from chromaffin cells in the adrenal medulla (phaeochromocytoma) or extra-adrenal chromaffin tissue (paraganglioma). Although they share overlapping clinical presentations, PPGLs demonstrate marked heterogeneity in biochemical secretion, genetic background, and clinical behaviour. Phaeochromocytomas often secrete both noradrenaline and adrenaline, whereas paragangliomas do not typically secrete adrenaline and can sometimes be non-secretory (1).

Phaeochromocytomas are typically benign (75-95%), whereas paragangliomas carry a higher risk of malignancy, with rates reported as high as 50% depending on anatomical location and genetic background (2, 3). Differentiating between benign and malignant disease remains challenging, even on histological assessment following surgical resection, as there are no established markers predicting malignant behaviour with absolute certainty. Histological features such as mitotic activity, cellular atypia, or neurovascular invasion lack consistent predictive value, with the only reliable indicator for malignancy being synchronous or metachronous metastases (4). These diagnostic challenges have been reflected in the 2022 WHO classification of endocrine tumours, defining PPGL tumours as malignant only in the presence of metastasis; otherwise, tumours are classified as localised (5).

With the recent expansion of genetic testing, it is now recognised that only 60% of PPGLs are truly sporadic, while the remaining 40% occur in the setting of germline mutations and syndromes. This figure is expected to rise in the coming years. Familial PPGLs include mutations and syndromes: multiple endocrine neoplasia type 2 (MEN2) due to RET proto-oncogene mutation, von Hippel-Lindau (VHL), succinate dehydrogenase subunits (SDHx -A, B, C and D subunits) (6), neurofibromatosis type 1 (NF1) and other less common mutations such as fumarate hydratase (FH) mutation causing mosaic fumarate hydratase syndrome and Beckwith-Wiedemann Syndrome (BWS) (7, 8). SDHB tumours are known to be associated with more aggressive clinical behaviour and a higher metastatic risk (9).

The mainstay of treatment is surgery; however, patients with metastatic or inoperable disease may benefit from molecular radiotherapy using ^131^I-mIBG and ^177^Lu-DOTATATE (10). In our previous study, we found that metastases were significantly more frequent in patients with paragangliomas compared to those with phaeochromocytomas (11). Metastases frequently involve lymph nodes, bone, liver, and lungs, and may be missed on initial cross-sectional imaging (12).

Patients with a biochemical diagnosis of PPGLs will typically undergo a combination of anatomical and/or functional imaging preoperatively for primary tumour confirmation, to identify single or multiple lesions, and metastatic disease. CT is considered the first-line imaging modality offering a high sensitivity of close to 90%. MRI is preferred in certain situations, including paediatric patients, pregnancy, patients with germline mutations who require frequent surveillance scans, and contrast allergies (3); however, both CT and MRI have low specificity rates in confirming PPGL tumours (13).

Several functional imaging modalities are available, each interrogating different biological properties of PPGL. Historically, ¹²³/¹³¹I-mIBG scintigraphy has been widely used to assess noradrenaline transporter expression, with reported sensitivities ranging from 56–88% (14). More recently, PET-based tracers have gained prominence. ^68^Ga-DOTATATE PET/CT targets somatostatin receptor expression and is valuable for theranostic decision-making, while ^18^F-FDG PET reflects tumour glucose metabolism and has been associated with dedifferentiation, aggressive behaviour, and SDHB-related disease. Despite their complementary roles, there remains no consensus regarding the optimal first-line functional imaging approach in PPGL, particularly for staging and identification of biologically aggressive disease.

In this study, we compared three commonly used functional imaging modalities—^18^F-FDG PET CT/MRI, ^68^Ga-DOTATATE PET/CT, and ^123^I-mIBG SPECT/CT—within the same patients with confirmed localised and metastatic PPGL. By correlating imaging findings with biochemical profiles and germline mutation status, we aimed to evaluate the extent to which functional imaging reflects tumour phenotype and to define a pragmatic FDG-first functional imaging approach for staging and risk stratification in PPGL.

## Methods

### Study design and participants

This was a retrospective, cross-sectional, within-patient diagnostic accuracy study conducted at a tertiary referral centre. All patients underwent baseline imaging at University College London Hospital in London, United Kingdom, between 2012 and 2023, including conventional MRI or CT for anatomical localisation. In addition, a subset of patients underwent the triple functional assessment consisting of ^18^F-FDG PET, ^68^Ga-Dotatate PET/CT, and ^123^I-mIBG SPECT/CT.

Patients were either referred for adjuvant or molecular radiotherapy following a pre-existing diagnosis of metastatic PPGL or were newly diagnosed cases of metastatic/unresectable PPGL at our institution. The study cohort comprised only patients with abdominal phaeochromocytomas and paragangliomas, and head and neck paragangliomas were not included. Diagnostic confirmation of metastatic PPGL was based on predefined reference standards, such as histological confirmation (if available), unequivocal anatomical progression on imaging studies, and/or typical clinical and biochemical picture with an MDT discussion.

**Figure 1:**
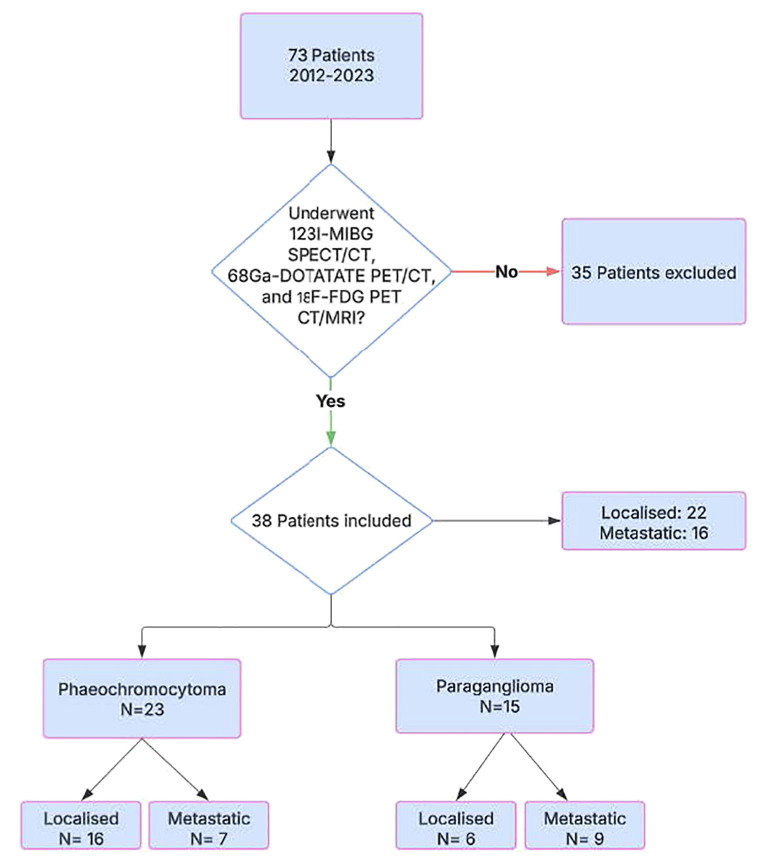
STARD flow diagram of patient selection, exclusion due to incomplete imaging, and analytic cohort formation for the within-patient comparative functional imaging study.

Data were collected for 73 consecutive patients; 38 patients who underwent all three functional imaging modalities comprised the analytic cohort, while 35 were excluded due to incomplete imaging. Patients were categorised as having localised or metastatic disease (**Figure 1**).

Metastatic cases included those diagnosed *de novo* at UCLH and those referred from external centres for further evaluation or therapy. Variables analysed included diagnostic findings, hormonal secretion profiles (plasma normetanephrine, metanephrine, and 3-methoxytyramine [3MT]), genetic mutations, and clinical outcomes. A secondary objective was to examine the relationship between metanephrine levels and tumour uptake on ^18^F-FDG PET CT/MRI.

### Imaging protocol

All imaging was performed according to local protocols aligned with European Association of Nuclear Medicine (EANM) guidelines (15).

Weight-based adjustment of injected activity was adopted to optimise radiation exposure from the respective tracers. For paediatric patients, scaling of injected activity was performed as per the EANM paediatric dosage card (15).

For ^123^I-mIBG imaging, medications known to interfere with mIBG uptake were reviewed and withheld as appropriate. Oral potassium iodide was administered prior to imaging for thyroid blockade. Imaging acquisition was performed at approximately 4 hours and 24 hours following tracer administration in accordance with local institutional protocols. SPECT/CT acquisition was performed over the abdomen, with additional anatomical coverage as clinically indicated.

For ^18^FDG, PET/CT or PET/MRI was selected for each patient based on scanner availability. Patients fasted for at least 6 hours prior to imaging and insulin for 4 hours when applicable. For both FDG and Dotatate imaging, tracer time was 60 minutes. PET acquisition was performed from skull base to mid-thigh, with low-dose non-contrast CT acquired for attenuation correction and anatomical localisation.

Scintigraphy and SPECT/CT studies were acquired on GE cameras systems (GE Healthcare Technologies, Milwaukee, USA), including Infinia Hawkeye or GE D670 platforms. PET/CT studies were performed on GE and Siemens systems (Siemens Healthineers, Erlangen, Germany) including GE Discovery VCT64, D710, and Siemens Biograph Vision scanners. Internal calibration and quality assurance procedures were maintained to optimise quantitative consistency across scanners. Standardised uptake value (SUV) measurements for ^18^F-FDG PET CT/MRI adhered to EARL accreditation standards applicable at the time of acquisition (15–18).

### Imaging interpretation and outcome definitions

A subgroup analysis included patients with histologically confirmed PPGL. The additional analysis included patients diagnosed with either localised or metastatic PPGL, where the primary outcome measure was per-patient lesion detection, defined as visually and quantitatively positive uptake—greater than background liver uptake—on a given functional scan, measured per patient.

SUV_max_ was measured in the suspected/confirmed lesion across all imaging time points on FDG and Dotatate scans; SUV was not measured on mIBG SPECT scans. The objective was to determine the optimal SUV measurement by identifying the highest SUV_max_ while using SUV_max_ of the liver as the reference background, then labelling the uptake as positive or negative.

The triple functional scans of preoperative or recurrent phaeochromocytomas and paragangliomas were reviewed confirming primary lesion or metastatic lesions (including bones, viscera, or lymph nodes), then the SUV measurement on FDG and Dotatate of the lesion was recorded and compared with liver uptake. A lesion was classified as metastatic after a consultant radiologist’s report demonstrating radiological characteristics similar to the primary lesion and confirmed by multidisciplinary team (MDT) review along with a similar or concordant response of the metastatic lesion to treatment on follow-up imaging.

Patients were classified as scan-positive if the identified lesion met the functional imaging criterion for PPGL and was confirmed on histology, cross-sectional imaging/clinical follow-up. In patients with metastatic disease at least one lesion site was confirmed positive on histology or unequivocal radiology and clinical evolution.

### Cost of imaging

Approximate functional imaging costs were estimated using the NHS tariff data (North East Central London, 2022/23). Tariff’s assumptions scans’ cost varying ±20%. Estimated costs were £2,700 (range £2160-£3240) for ^123^I-mIBG SPECT/CT; £948 (range £758-£1137) for^18^F-FDG PET CT/MRI (MRI component higher; £750-1000), and £2,807 (range £2245-£3368) for ^68^Ga-DOTATATE PET/CT. An additional estimated cost of approximately £1,000 was applied when scans were performed under general anaesthesia. These figures are presented for illustration comparison only.

### Statistical analysis

The detection rates were assessed using descriptive statistics, with per-patient detection as the primary performance measure. The primary outcome was comparison of per-patient detection rates across imaging modalities. Positive or negative lesion uptake was determined by comparing lesion uptake with background liver activity and summarised cross tumour subgroups using SPSS Statistics, version 26 (IBM Corp.). A lesion is considered positive if the uptake exceeds background liver activity. A two-sided p-value < 0.05 was considered statistically significant.

Plasma biochemical markers were summarised as medians (range), owing to non-parametric data distribution, while SUV-based analysis were reported as means (± SD). Correlations between SUVmax and plasma biomarkers were assessed using spearman’s rank correlation coefficient, given the non-parametric distribution of biochemical variables.

Traditional diagnostic accuracy metrics such as sensitivity, specificity, predictive values, and area under the receiver operating characteristic curve were not calculated, as the study employed a within-patient comparative design using a composite, non-independent reference standard. In this context, per-patient detection rate was considered the most clinically relevant and methodologically appropriate performance measure.

## Results

### Patients characteristics

Thirty-eight patients underwent triple functional imaging, of those twenty-three patients (60.5%) had phaeochromocytoma and 15 (39.5%) had paragangliomas. Eighteen patients were female (47.4%), and 20 were male (52.6%). The median age at diagnosis was 41 years (range 11–81 years). Plasma metanephrine measurements were available for all patients. Median plasma concentrations at presentation were 6,329 pmol/L (range 10–70,153 pmol/L) for normetanephrine, 447 pmol/L (range 0.06–29,168 pmol/L) for metanephrine, and 230 pmol/L (range <100–1,340 pmol/L) for 3-methoxytyramine.

Genetic testing was performed in 21 patients (55%). No pathogenic mutation was identified in 9 patients (23.7%). SDHB mutations were detected in 6 patients (15.8%), VHL mutations in 3 patients (7.9%), and single cases (2.6% each) were associated with RET mutation, mosaic fumarate hydratase deficiency, and Beckwith–Wiedemann syndrome. Seventeen patients (44.7%) did not undergo genetic testing (**Table 1**).

**Table 1:** Patient demographics and frequency analysis.

Characteristics	Variables	Frequency	Percent
Sex	Female	18	47.4%
	Male	20	52.6%
Diagnosis	Phaeochromocytoma	23	60.5%
	Paraganglioma	15	39.5%
^18^F-FDG PET CT/MRI	Positive	36	94.7%
	Negative	2	5.3%
^68^Ga-DOTATATE PET/CT	Positive	35	92.1%
	Negative	3	7.9%
^123^I-mIBG SPECT/CT	Positive	34	89.5%
	Negative	4	10.5%
Mutation	Not performed	17	44.7%
	Performed:		
	No pathogenic mutation	9	23.7%
	SDHB	6	15.8%
	VHL	3	7.9%
	Mosaic Fumarate Hydratase	1	2.6%
	Beckwith-Wiedemann Syndrome	1	2.6%
	RET mutation	1	2.6%
Tumour Behaviour	Localised	22	57.9%
	Metastatic	16	42.1%

Tumours were classified as localised or metastatic; 22 patients (57.9%) had local disease, and 16 (42.1%) had metastatic disease (**Table 1**).

### Per-patient detection across functional imaging modalities

Among the 38 patients who underwent triple functional imaging, per-patient detection rates were 36/38 (94.7%) for ^18^F-FDG PET, 35/38 (92.1%) for ^68^Ga-DOTATATE PET/CT, and 34/38 (89.5%) for ^123^I-mIBG SPECT/CT.

In patients with phaeochromocytoma (n = 23), detection was highest with FDG (21/23, 91.3%), followed by Dotatate (20/23, 87.0%) and mIBG (20/23, 86.9%). In all patients with paraganglioma (n = 15), both FDG and DOTATATE demonstrated detection in all cases (15/15, 100%), while mIBG was positive in 14/15 patients (93.3%). Across all analysed lesions, the observed SUVmax values ranged from 1.1 to 39.0 for 18F-FDG PET CT/MRI and from 3.3 to 69.0 for 68Ga-DOTATATE PET/CT.

Among patients with metastatic phaeochromocytoma (n = 7), FDG demonstrated detection in all cases (7/7, 100%), compared with 5/7 (71.4%) for DOTATATE and 3/7 (42.9%) for mIBG. In metastatic paraganglioma (n = 9), all three modalities demonstrated detection in all patients.

When metastatic disease was analysed by lesion type, primary malignant lesions (n = 3) demonstrated uptake on FDG and mIBG in all cases (3/3, 100%) and on DOTATATE in 2/3 (66.7%). In metastatic lesions (n = 13), detection was highest with FDG (13/13, 100%), followed by DOTATATE (12/13, 92.3%) and mIBG (11/13, 84.6%) (**Table 2**). Paired comparison of detection rates across modalities did not reach statistical significance (Cochran’s Q test).

**Table 2:** Lesion uptake per-patient in each functional scan.

Functional scan	Lesion Detection Rate (n/N, %, 95% CI)					
	Primary (non metastatic) phaeochromocytoma N: 16	Primary (non metastatic) paraganglioma N: 6	Metastatic phaeochromocytoma N: 7	Metastatic paraganglioma N: 9	In metastatic disease	
					Primary lesion N: 3	Metastatic lesion N: 13
_123_I-MIBG SPECT/CT	16/16 (100%, 95% CI 59.0–100.0)	5/6 (83.3%, 95% CI 68.1–99.8)	3/7 (42.9%, 95% CI 9.9–81.6)	9/9 (100%, 95% CI 66.4–100.0)	3/3 (100%, 95% CI 29.2–100.0)	11/13 (84.6%, 95% CI 54.6–98.1)
_18_F-FDG PET CT	14/16 (87.5%, 95% CI 72.0–98.9)	6/6 (100%, 95% CI 59.0–100.0)	7/7 (100%, 95% CI 59.0–100.0)	9/9 (100%, 95% CI 66.4–100.0)	3/3 (100%, 95% CI 29.2–100.0)	13/13 (100%, 95% CI 75.3–100.0)
_68_Ga-DOTATATE PET/CT	15/16 (93.8%, 95% CI 66.4–97.2)	6/6 (100%, 95% CI 59.0–100.0)	5/7 (71.4%, 95% CI 29.0–96.3)	9/9 (100%, 95% CI 66.4–100.0)	2/3 (66.7%, 95% CI 9.4–99.2)	12/13 (92.3%, 95% CI 64.0–99.8)
p-value (Cochran’ s Q):	0.368	0.368	0.174	Not applicable		

N, number of patients with positive lesions.

**Figure 2:**
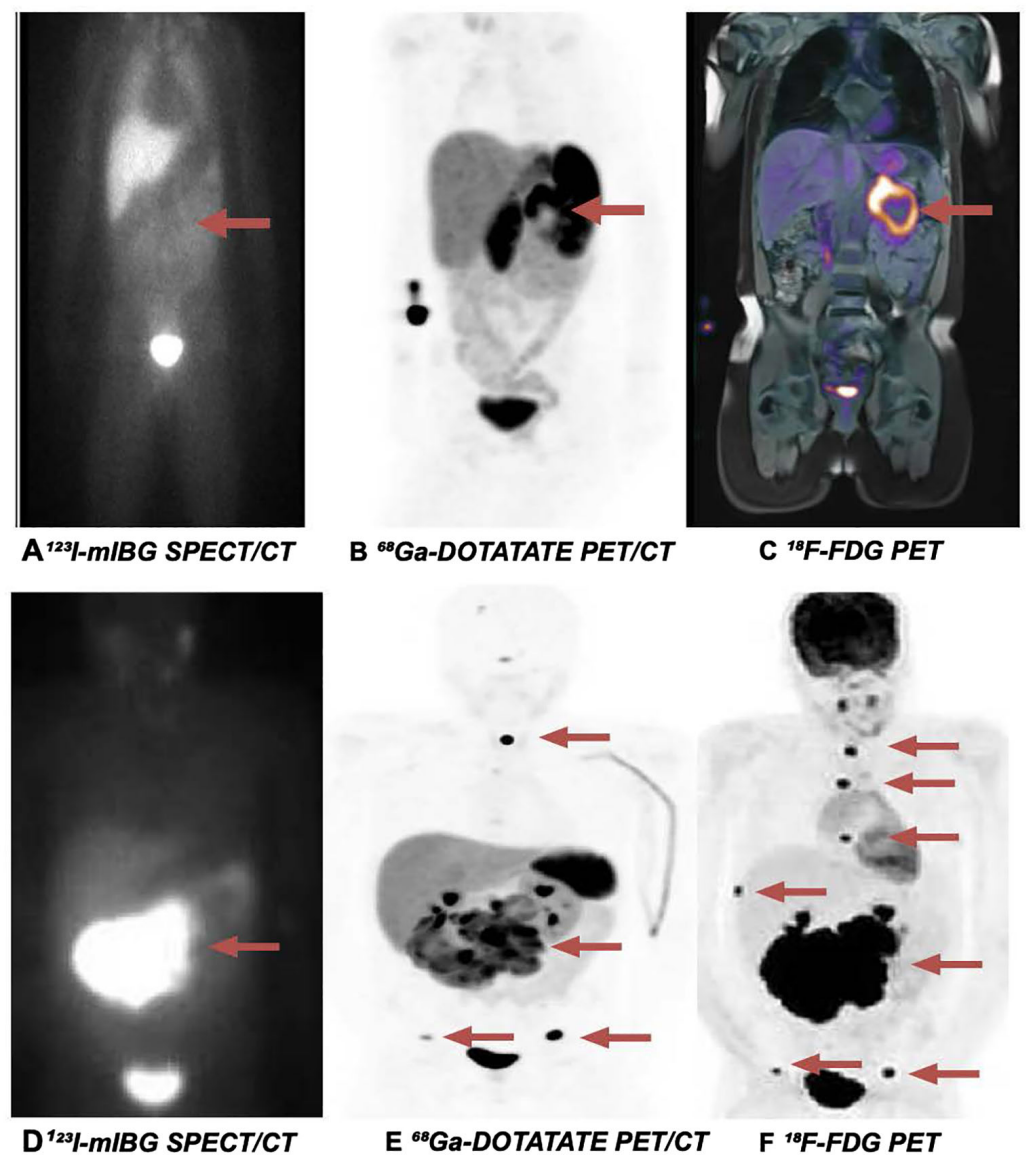
Comparison between three scanning modalities in benign and malignant disease **(A–C)** A patient with a benign left adrenal phaeochromocytoma, **(A)**^123^I-mIBG SPECT/CT shows no radiotracer uptake in the lesion, **(B)** ^68^Ga-DOTATATE PET/CT reveals positive uptake in the left adrenal mass, consistent with somatostatin receptor expression, **(C)** ^18^F-FDG PET CT/MRI demonstrates avid uptake, indicating high metabolic activity with central photopenia suggestive of tumour necrosis and indicative of heterogeneous update. **(D–F)** A second patient with an abdominal paraganglioma showing positive uptake across all modalities, **(D)**^123^I-mIBG SPECT/CT demonstrates uptake confined to the primary lesion without evidence of metastatic disease, **(E)** ^68^Ga-DOTATATE PET/CT with red arrows showing areas of metastatic bone disease in skull, spine and pelvis without corresponding metastatic disease on cross sectional imaging **(F)** ^18^F-FDG PET CT demonstrates primary lesion uptake SUV max 26.9 with the greatest metastatic disease burden including (spinous process T1 SUV max 17.7, T7 SUV max 9.1, in addition to right eight rib and bilateral pelvic bones.

Representative imaging findings are shown in **Figure 2**.

### Correlation between functional imaging uptake and biochemical markers

SUVmax values from FDG and DOTATATE PET imaging were analysed in relation to plasma normetanephrine, metanephrine, and 3-methoxytyramine concentrations. FDG SUVmax demonstrated a moderate positive correlation with plasma 3-methoxytyramine (Spearman’s r = 0.56, p = 0.003), whereas no significant correlations were observed between FDG uptake and plasma normetanephrine or metanephrine levels. DOTATATE SUVmax showed no significant correlation with any plasma biochemical marker (**Table 3**).

**Table 3:** Correlation between SUV_max_ and plasma biomarkers.

Function scan suvmax value	Blood marker	Correlation (r)	p-value	SUV mean	SUV SD	Blood mean	Blood SD
^18^F-FDG PET CT/MRI	Plasma Normetanephrines	-0.03	0.8785	8.77	8.03	11041.9	15227.3
	Plasma Metanephrines	-0.26	0.2181	8.77	8.03	3180.9	5369.9
	Plasma 3MT	0.56	0.0034	8.77	8.03	288.5	305.0
_68_Ga-DOTATATE PET/CT	Plasma Normetanephrines	-0.03	0.8924	22.05	13.42	11041.9	15227.3
	Plasma Metanephrines	0.04	0.8622	22.05	13.42	3180.9	5369.9
	Plasma 3MT	0.04	0.8653	22.05	13.42	288.5	305.0

### Genotype-specific detection

Among patients who underwent genetic testing, FDG demonstrated per-patient detection in all patients with SDHB mutations (6/6) and VHL mutations (3/3), and in 8/9 (88.9%) patients without an identified pathogenic mutation. DOTATATE demonstrated detection in all patients with SDHB mutations and in all mutation-negative patients, while mIBG demonstrated detection in all patients with VHL mutations and in all mutation-negative patients. None of the genotype-specific comparisons reached statistical significance, reflecting limited subgroup sizes (**Table 4**).

**Table 4:** Genetic association to lesion uptake.

Scan	Mutation				Fisher’s Exact Test	P value
	scan result	SDHB	VHL	Negative		
_18_F-FDG PET CT/MRI	Negative	0	0	1 (11.1%)	1.408	1
	Positive	6 (100.0%)	3 (100.0%)	8 (88.9%)		
_68_Ga-DOTATATE PET/CT	Negative	0	1 (33.3%)	0	3.606	0.167
	Positive	6 (100.0%)	2 (66.7%)	9 (100.0%)		
_123_I-mIBG SPECT/CT	Negative	1 (16.7%)	0	0	2.219	0.5
	Positive	5 (83.3%)	3 (100.0%)	9 (100.0%)		

## Discussion

This study is among the few to date analysing the triple functional imaging assessment within the same patients, alongside plasma metanephrine profiles and germline mutation data. Historically, ^123^I-mIBG was regarded as the reference functional scan in PPGL; more recently, PET-based tracers have increasingly challenged this paradigm, particularly in advanced or biologically aggressive disease.

Our findings demonstrated that ^18^F-FDG PET CT/MRI had the highest overall detection rate and outperformed other modalities in metastatic disease and high-risk genetic subgroups. Detection rates were analysed on a per-patient basis, aligning with real-world clinician decision-making rather than lesion-level enumeration. These results provide practical guidance for clinicians involved in PPGL care, including surgeons and endocrinologists, and nuclear medicine specialists, when selecting the first-line functional modality.

In this study, ^18^F-FDG PET/CT or PET/MRI demonstrated consistently higher per-patient lesion detection across PPGL subtypes and showed the strongest association with biochemical and genetic markers of aggressive disease. These findings support an FDG-first functional imaging approach for diagnosis and staging, which may reduce the need for multiple functional studies and facilitate timely clinical decision-making.

In contrast, ^123^I-mIBG scintigraphy retains an important role in identifying patients eligible for ¹³¹I-mIBG molecular radiotherapy, while ^68^Ga-DOTATATE PET/CT remains valuable for confirming somatostatin receptor (SSTR) expression and informing theranostic considerations. Taken together, a selective and sequential use of functional imaging modalities may improve diagnostic efficiency and support personalised management strategies in PPGL.

Earlier comparative work favoured Dotatate over FDG due to its higher lesion-to-background contrast and somatostatin receptor specificity (3, 19, 20). However, subsequent work has shown that FDG demonstrates superior diagnostic performance in metastatic PPGL, SDHB-associated disease (21), and even in non-SDHx, localised tumours (2, 14, 22, 23). Timmers et al. reported that one-third of PPGL patients had false-negative mIBG scans, whereas FDG demonstrated significantly improved sensitivity in these cases (14). Furthermore, FDG offers key advantages over both mIBG and DOTATATE, including faster acquisition, better image quality, and true quantitative capabilities (24).

In the present cohort, FDG demonstrated the highest overall per-patient detection rate (94.7%), followed by Dotatate (92.1%) and mIBG (89.5%). FDG achieved 100% detection in all metastatic PPGLs, including both metastatic phaeochromocytomas and paragangliomas. In contrast, mIBG showed limited uptake in advanced disease, with detection in only 43% of metastatic phaeochromocytomas, reinforcing its reduced sensitivity in aggressive tumour phenotypes. The inclusion of patients undergoing both initial diagnostic evaluation and assessment for metastatic disease reflects real-world referral patterns in PPGL, particularly in paragangliomas and high-risk genotypes, such as SDHB. While the temporal onset of metastatic disease could not always be established, FDG detected metastatic involvement whenever present at the time of imaging, supporting its utility for diagnosis and staging in high-risk disease.

Published thresholds suggest that PPGL is unlikely when the SUVmax is <2.1 for FDG and <4.3 for DOTATATE, and likely when >10.8 and >24.5, respectively (14). Although Dotatate demonstrated higher SUV_max_ values in both primary and metastatic lesions, this did not translate into significantly improved detection in metastatic disease. The heterogeneity of somatostatin receptor expression may account for variable uptake and reduced reliability in aggressive or dedifferentiated tumours. It is also worth mentioning that there could be variation in interpreting FDG uptake patterns. Tumour necrosis, which is a common histopathological feature in phaeochromocytomas, especially in larger or more aggressive lesions, results in metabolically inactive areas and therefore does not take up FDG, leading to heterogeneous uptake. As a result, the pattern of FDG avidity may vary from diffuse uptake across the lesion to rim-like peripheral uptake, which is usually preserved (15).

A key strength of FDG lies in its biological correlation with tumour aggressiveness. We observed a moderate but statistically significant correlation between FDG SUV_max_ and plasma 3-methoxytyramine (3MT) levels (r = 0.56, p = 0.0034), a biomarker linked to dopamine-secreting, extra-adrenal, and metastatic tumours (25). In contrast, no significant correlation was observed between SUV_max_ and normetanephrine or metanephrine, nor with SUV_max_ on Dotatate. FDG also outperformed other modalities in genetically stratified subgroups. It showed 100% detection in patients with SDHB (26) and VHL mutations—mutations known to activate the pseudohypoxia pathway via HIF1α stabilisation and aerobic glycolysis (22, 27–29). Although not statistically significant, likely due to sample size, this finding aligns with earlier reports demonstrating high FDG avidity and metastatic risk in SDHB-mutated PPGL (30, 31).

Beyond diagnostic performance, FDG offers substantial advantages in global accessibility and operational efficiency. FDG PET is more widely available and less resource-intensive than Dotatate or mIBG, particularly within publicly funded healthcare systems. In the UK, ^18^F-FDG PET CT is widely commissioned, whereas Dotatate availability is constrained by generator cost, while mIBG involves time-intensive protocols and restricted radio pharmacy supply (21). These logistical factors, together with acceptable radiation exposure across all modalities, further support the pragmatic use of FDG as the first-line functional imaging.

In the UK, NHS and independent sector pricing typically places FDG PET CT at £750–£1,000 per scan, with MRI component carrying higher costs but used in selected cases, whereas Dotatate is less widely available and incurs higher operational costs due to generator requirements, isotope shelf-life, and limited manufacturing infrastructure. The radiation exposure associated with all three imaging modalities—FDG, Dotatate, and mIBG—remains within internationally accepted diagnostic reference levels. Typical effective doses range from 5–14 mSv, depending on scan protocol, which is justified by the diagnostic and therapeutic importance in PPGL. Moreover, the use of ^18^F-FDG PET MRI scans, as employed in this study, offers the additional benefit of reducing cumulative radiation exposure by eliminating the CT component at the expense of cost. According to the IAEA IMAGINE database (32), ^18^F-FDG PET is available in over 140 countries, while ^68^Ga-DOTATATE PET/CT is used in fewer than 50, and ^123^I-mIBG imaging is confined to specialised centres. The acquisition of mIBG and Dotatate often requires complex coordination across nuclear medicine, radio pharmacy, and regulatory agencies, leading to delays or cancellations. These logistical barriers are compounded by frequent shortages and infrastructure constraints, particularly in low- and middle-income countries. In contrast, FDG PET’s short half-life, widespread cyclotron production, and standardised protocols support faster access and broader implementation.

Given its superior detection rates in PPGL, alignment with tumour biology, and widespread availability, we advocate for FDG as the preferred first-line functional imaging modality for PPGL. Nevertheless, it is important to emphasise that while FDG is optimal for diagnosis and staging, it currently lacks an associated therapeutic pathway. In contrast, both mIBG and Dotatate provide critical theranostic value. In addition, our data did not include head and neck paragangliomas, a subgroup in which Dotatate typically demonstrates superior performance. Uptake on ^123^I-mIBG scans is a prerequisite for consideration of ^131^I-mIBG therapy, and somatostatin receptor expression, confirmed by ^68^Ga-DOTATATE PET/CT, is essential for eligibility for peptide receptor radionuclide therapy (PRRT) with ^177^Lu-DOTATATE. Thus, although FDG may suffice for initial work-up and follow-up, additional imaging with mIBG or Dotatate remains necessary in patients for whom targeted radionuclide therapy is being considered. While Dotatate remains valuable in receptor-targeted diagnostics or for PRRT planning, and ^123^I-mIBG retains relevance in selecting candidates for ^131^I-mIBG therapy, these modalities should be reserved for select indications rather than routine staging.

In the UK NHS context, ^131^I-mIBG therapy is available under the National Health Service (NHS) for patients with PPGL who demonstrate radiotracer uptake on diagnostic ^123^I-mIBG imaging. There is, however, a significant shortage of supply in Europe, which may make treatment less possible. To complicate matters, the use of ^177^Lu-DOTATATE is not commissioned by the NHS for phaeochromocytomas and paragangliomas, and is limited to clinical trial settings only. This NHS differential funding landscape supports that when both modalities are available, a diagnostic ^123^I-mIBG scan is favoured to assess eligibility for ^131^I-mIBG therapy, while Dotatate may be performed in cases where mIBG is negative or inaccessible. Hence, functional imaging and therapeutic sequencing are structured to prioritise mIBG-directed treatment where possible (33, 34).

Emerging agents (e.g., ^18^F-mFBG) may further refine specificity and workflow and could shift practice as availability and commissioning evolve. Until then, an FDG-first strategy, complemented by targeted mIBG/DOTATATE for theranostic planning, offers a cost-aware, patient-centred, and biologically aligned pathway (35, 36). Beyond initial staging, FDG PET may also have utility in treatment response assessment and disease monitoring, with recent studies suggesting that volumetric FDG PET parameters may provide prognostic information following radionuclide therapy in advanced PPGL (37).

**Figure 3:**
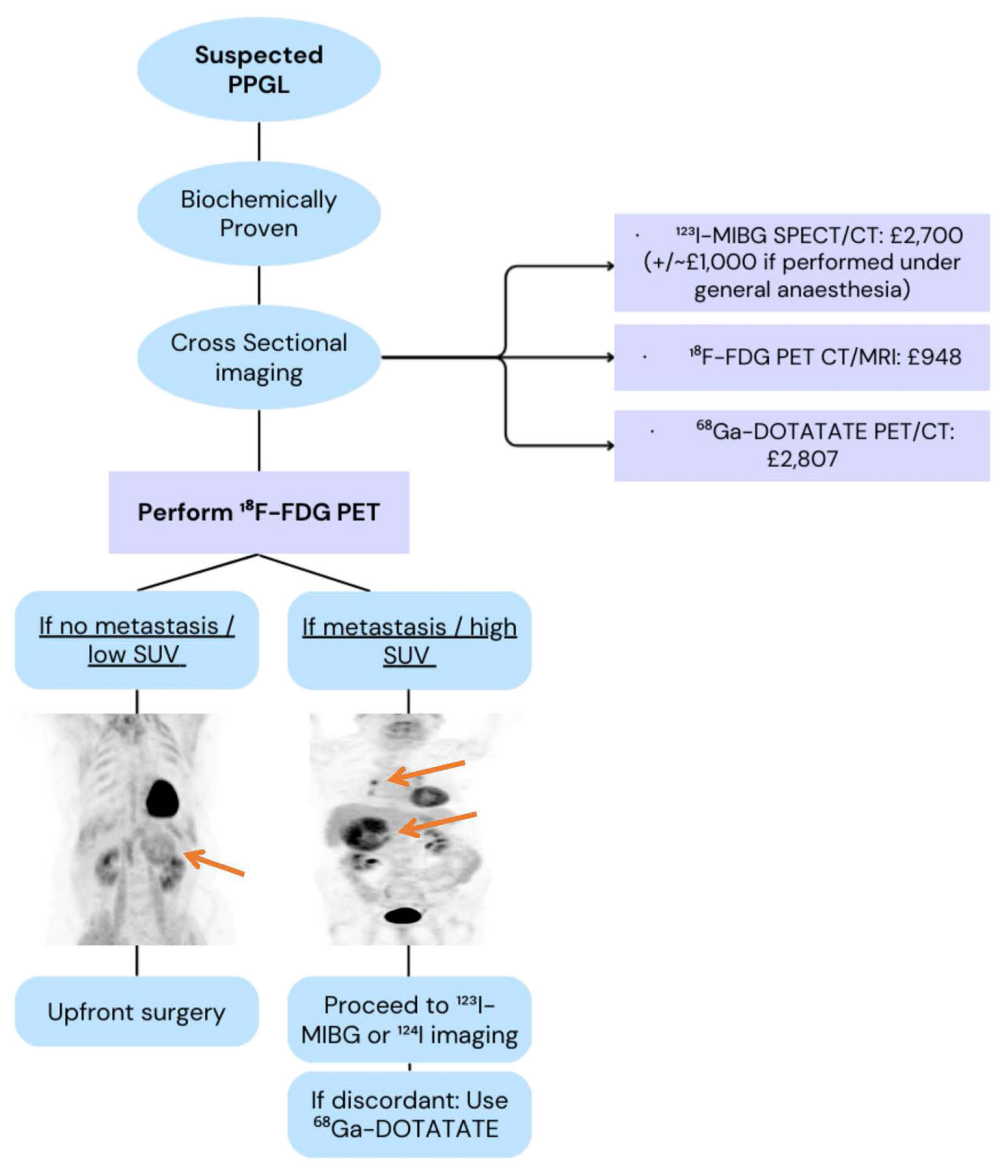
Proposed diagnostic and therapeutic algorithm for functional imaging in phaeochromocytoma and paraganglioma (PPGL). The algorithm integrates availability, cost, and therapeutic implications of different nuclear medicine modalities within the NHS. ^18^F-FDG PET CT/MRI is recommended as the primary functional imaging modality given its high sensitivity, lower cost, and accessibility. ^68^Ga-DOTATATE PET/CT has a useful diagnostic role in somatostatin receptor–positive disease but currently lacks a funded therapeutic counterpart in the NHS, as ^177^Lu-DOTATATE is not commissioned for PPGL. In contrast, ^123^I-mIBG scintigraphy, while less sensitive, retains value in patients with positive uptake because it can guide and enable ^131^I-mIBG radionuclide therapy, which is NHS-funded.

Based on the comparative detection performance observed in this study, the recognised biological heterogeneity of PPGL (38), and current therapeutic commissioning, we propose a pragmatic functional imaging pathway for patients with suspected PPGL (**Figure 3**).

A key strength of this study is the within-patient comparative design, which allowed direct head-to-head evaluation of three commonly used functional imaging modalities while minimising confounding related to tumour biology, disease burden, and patient-specific factors. The inclusion of triple-modality imaging in the same patients is uncommon in PPGL research and provides a robust framework for comparative performance assessment. In addition, integration of functional imaging findings with plasma biochemical profiles and germline mutation status enabled biologically informed interpretation of imaging performance, particularly in aggressive and high-risk disease phenotypes. The use of per-patient detection as the primary outcome reflects real-world clinical decision-making and enhances the translational relevance of the findings in tertiary referral practice.

This study has limitations. Although 73 consecutive patients with suspected PPGL were identified, the comparative analysis was restricted to the 38 patients who underwent all three functional imaging modalities. This requirement may have enriched the analytic cohort for patients with more complex disease, metastatic involvement, or those being assessed for theranostic decision-making, potentially limiting population representativeness. As a result, the findings may not be directly generalisable to all patients with PPGL, particularly those with straightforward localised disease. The retrospective design may predispose to selection bias. The sample size is also small due to the rarity of PPGL, limiting statistical power for subgroup analysis. Heterogeneity in disease stage and timing between imaging studies may have influenced tracer uptake and lesion detectability. Genetic testing was not available in all patients, limiting interpretation of genotype-specific imaging correlations. In addition, imaging availability, healthcare commissioning, and local diagnostic pathways vary internationally, and therefore the proposed FDG-first approach may not be directly generalisable to all healthcare systems or PPGL subtypes. Finally, incorporation bias cannot be entirely excluded, as functional imaging findings may have contributed to the composite reference standard.

In conclusion, ^18^F-FDG PET CT/MRI demonstrated the highest per-patient lesion detection across PPGL phenotypes and showed the strongest association with biochemical and genetic markers of aggressive disease. These findings support an FDG-first functional imaging strategy for diagnosis and staging in PPGL. While ^123^I-mIBG SPECT/CT and ^68^Ga-DOTATATE PET/CT remain essential for theranostic assessment and selection for radionuclide therapy, their use is best reserved for selected indications rather than routine staging. A selective, sequential imaging approach aligned to tumour biology and therapeutic intent may streamline diagnostic pathways and support precision-guided management in PPGL.

## Data Availability

The raw data supporting the conclusions of this article will be made available by the authors on reasonable request.
